# Developing and testing a reflection method for implementation of the informal care guideline in community nursing: Design‐based research

**DOI:** 10.1111/jan.16156

**Published:** 2024-03-21

**Authors:** Nicole Vullings, Marjo Maas, Marian Adriaansen, Hester Vermeulen, Philip van der Wees, Maud Heinen

**Affiliations:** ^1^ Institute of Nursing Studies HAN University of Applied Sciences Nijmegen The Netherlands; ^2^ IQ Health Science Department, Research Institute for Medical Innovation Radboud University Medical Center Nijmegen The Netherlands

**Keywords:** community care, nursing home care, practice nursing, reflective practice, research implementation

## Abstract

**Aim:**

To develop a reflection method for community nurses and certified nursing assistants to support the implementation of the Dutch Informal Care guideline in daily care.

**Design:**

Design‐based research.

**Methods:**

A design group and four test groups of community nurses and nursing assistants were formed to develop a reflection method that aligns with the needs and preferences of its end‐users. The design and test group meetings were video recorded. The video data were iteratively discussed and analysed thematically to adapt and refine the method and to identify its key features.

**Results:**

A final reflection method was developed. Five main themes were identified from the analysis: the group, reflective triggers, knowledge about the guidelines, the coach and preconditions. The themes are linked to nine key features representing the building blocks of the reflection method. The key features are group size, participants with different (educational) backgrounds, pairs of participants, expressing thoughts, video feedback, reflection game, making the connection with the guideline, coaching as a process facilitator and meeting organizational and contextual conditions for implementation.

**Conclusion:**

An evidence‐ and practice‐based reflection method for community nurses and certified nursing assistants is developed to support the implementation. By involving community nurses and certified nursing assistants, the method closely matches their needs and preferences. Critical elements of the reflection method are a game element, video feedback and working in pairs in a group of participants from different (educational) backgrounds. Guidance is needed to make the transfer from theory to practice.

**Impact:**

A reflection method for community nurses and certified nursing assistants was developed to enhance care work according to guideline recommendations, aiming to improve the care provided by informal caregivers.

**Report Method:**

The COREQ guideline was used.

**Patient or Public Contribution:**

This reflection method was developed in close collaboration with all stakeholders during the entire study.

## 1 INTRODUCTION

Nurses and certified nursing assistants are expected to work according to the principles of evidence‐based practice (EBP). This means that their decisions are based on a conscious consideration of available scientific knowledge, professional expertise and the desires and preferences of the client and their relatives. Guidelines are designed to facilitate EBP behaviour, with the aim of enhancing the quality of client care, reducing unwanted practice variations and promoting patient well‐being (Arts et al., 2016; Fischer et al., 2016; Flottorp et al., 2013). Clinical guidelines are based on systematic research of current evidence and offer graded recommendations that reflect best practices (Cassidy et al., 2021). Clinical guidelines have proven effective in improving health outcomes and processes of care (Gundersen, 2000). However, the implementation of clinical guidelines remains complex (Grol, 2001). Research has shown that on average 60% of care is consistent with existing evidence or clinical guideline recommendations (Braithwaite et al., 2020). The use of clinical guidelines in daily practice is challenging because nurses often are not aware of existing guidelines, and when they are, they consider them too extensive and not fully useful for individual patients (Magwood et al., 2020; Ovretveit et al., 2018). Moreover, they have limited time for quality‐improvement activities such as the implementation of guidelines (Arts et al., 2016; Fischer et al., 2016; Flottorp et al., 2013). In community nursing, an additional challenge is the solo nature of the profession. As a result, guideline recommendations are rarely used, and related interventions and tools are often not used (Correa et al., 2020; Fischer et al., 2016). To facilitate the use of guidelines for community nurses and certified nursing assistants, it is important to develop methods to promote the implementation of guidelines. In this study, the focus was on the Informal Care guideline. With the changes in healthcare, and the growing number of informal caregivers, this guideline was written to support community nurses and certified nursing assistants to prevent and diminish informal caregiver overload (V&VN, 2021).

## 2 BACKGROUND

Informal caregivers are defined as individuals providing unpaid care and assistance to a dependent person due to a disease, disability or health problem causing a loss of autonomy (Schulz & Tompkins, 2010). Informal caregiving is known to have benefits, like a sense of personal growth and improved interpersonal relationships (Cheng et al., 2012; Marino et al., 2017) but can also be very stressful and daunting (Chiao et al., 2015; Mohanty & Niyonsenga, 2019; Stratmann et al., 2021).

Due to the increase in the elderly population, the prevalence of chronic diseases, both the number and the importance of the role of the informal caregiver is growing (Hoad, 2002; Hussein & Manthorpe, 2014; Talley & Crews, 2007; WHO, 2023). Previous research has shown the crucial role of informal caregivers in long‐term care management and in reducing healthcare costs (Rabarison et al., 2018). In Europe, 20%–44% of all adults provide informal care to a relative (Verbakel, 2018). Governments across different countries over the world emphasize the need for informal caregiver support (Philp, 2001; van Dijk et al., 2013). In practice, this will require increased collaboration between informal caregivers and healthcare professionals.

Nurses and certified nursing assistants can have a crucial role in identifying, monitoring and preventing overload in informal caregivers and how they can better align formal and informal care. In practice, this appears to be challenging because nurses and certified nursing assistants often pay little attention to the informal caregiver while they are focusing on client care (V&VN, 2021). When attention is given to the informal caregiver, the literature shows that it lacks a structured approach, and nurses mainly base their actions on experience. On the other hand, informal caregivers are unlikely to seek help from nurses because they often do not see a solution for the overload or are reluctant to express concerns about their role as informal caregivers (V&VN, 2021). They take their caregiving responsibilities for granted—often their beloved ones—and fear potential consequences when discussing their role (de Boer et al., 2009; Zarzycki et al., 2022), for example, transfer to a nursing home.

To better support informal caregivers, and to prevent informal caregivers overload, the clinical Dutch guideline Informal Care was developed (V&VN, 2021). The purpose of this guideline is to provide guidance and tools for nurses and certified nursing assistants in their daily work, with substantiated recommendations on (1) how they can support the informal caregivers, (2) how they can identify, prevent, and monitor informal caregiver burden and (3) how to better coordinate formal and informal care.

A promising strategy to enhance adherence to guideline recommendations is to stimulate critical reflection and feedback on the care provided, and to promote continuous learning and improvement (Grol, 2001; Grol & Grimshaw, 2003; Ivers et al., 2012). Critical reflection allows for the discussion of complex and unfamiliar clinical situations in healthcare in which the conduct of guideline needs support (Rolfe, 2014). Reflective practice and performance feedback can enhance awareness and behaviour change regarding guideline adherence of healthcare professionals by giving meaning to their experiences, and understanding their strengths and weaknesses (Goulet et al., 2016; Maas et al., 2015; van Dulmen et al., 2014).

## 3 THE STUDY

### 3.1 Study aim

The aim of this study was to develop a reflection method for community nurses and certified nursing assistants to support the implementation of the Dutch Informal Care guideline (V&VN, 2021) in daily care.

### 3.2 Study design

A design‐based research approach was employed to develop the reflection method and to identify its key features. This design was chosen to optimally align with the different needs and abilities of certified nursing assistants and community nurses to implement guideline recommendations in daily practice. Design‐based research is increasingly gaining popularity in healthcare and welfare due to the complexity of care issues in the healthcare sector, as conducting research in the context and iteratively designing methods yields authentic and actionable knowledge (Armstrong et al., 2022; Przybilla et al., 2018). Design‐based research is focusing on developing an effective method or intervention and discovering new principles on what does and does not work by employing multiple iterations of developing, testing, evaluating and implementing in close collaboration with important stakeholders (Wang & Hannafin, 2005). Because of its flexible, iterative and, co‐creative approach, design‐based research is a suitable approach to develop a reflection method that works for both nurses and certified nursing assistants (van't Veer et al., 2020; Wang & Hannafin, 2005). This research was conducted in collaboration with community nurses and certified nursing assistants, who are considered co‐participants within the context of design‐based research. Co‐participants are seen as necessary in ‘helping to formulate the research questions’, ‘making refinements in the designs’ and ‘evaluating the impact of the reflection method’ (Armstrong et al., 2022).

The initial objective was to collaboratively design the reflection method, aiming to achieve the second goal of design‐based research, which encompasses intangible and theoretical outcomes (Armstrong et al., 2022). Additionally, this process aimed to improve the transferability of the reflection method to alternative guidelines or contexts, leading to the identification of key features inherent to the reflection method. Key features are the features of the reflection method that positively contribute to critical reflection on daily routines and support the transfer of guideline knowledge into daily practice (Greenhalgh et al., 2019; Grol & Grimshaw, 2003).

### 3.3 Study setting

This study is part of the SPARK project (Self & Peer Assessment to Reflect on Quality Standards). Within the SPARK project, reflection methods are developed for two guidelines, one process‐based guideline on nurse reporting (Vullings et al., Submitted) and one guideline related to direct client care (informal care), both in the community setting.

This study was conducted in the Netherlands between April 2022 and June 2023.

### 3.4 Participants and recruitment

The population for this research comprised community nurses, certified nursing assistants and one patient representative in the Netherlands. Initially, four regional home care organizations were invited to participate, of which two declined due to the COVID‐19 pandemic. Six community organizations were therefore additionally invited by email to participate in the study, of which agreed, giving four organizations altogether. The participating home care organizations were located in different regions across the Netherlands.

From the four participating home care organizations, a convenience sample of community nurses and certified nursing assistants was invited. The participants were contacted by email with an information letter and an informed consent document by the first author (NV). The patient representative was approached through an independent consultancy office that helps organizations facilitate equal cooperation between patients and healthcare professionals at an organizational level (IKONE, n.d.).

Each of the four test groups contained six community nurses and certified nursing assistants from four different organizations, and an internal coach to facilitate the reflection process during the test meetings. For this purpose, the coach was a trained community nurse. This study was conducted in two test phases. For each test phase, two of the four participating home care organizations were selected. Table 1 presents an overview of the participants, aims and intended outcomes for phase 1 and 2.

**TABLE 1:** Overview of the aims and outcomes for each phase.

Phase	Aim	Participants	Intended outcome
Design phase 1 (prototype)	To probe work formats and materials for the design group	Research group (*n* = 6)	Appropriate design methods and materials for the first prototype
	To identify what the reflection method should yield for the community nurses, certified nursing assistants and informal caregivers To identify key features of the reflection within a team or group of community nurses and certified nursing assistants	Design group and research group: Community nurses (*n* = 4), certified nursing assistants (*n* = 4), patient representatives (*n* = 1). Research group representatives (*n* = 3)	Desired yields for community nurses, certified nursing assistants and informal caregivers Key features for effective reflection and the use of the reflection method in clinical practice
	To develop different prototypes and associated working methods and to select one	Research group Representatives of the research group (*n* = 3) Research group (*n* = 6)	Prototype 1 of the reflection method
Test phase 1	To test and evaluate the first prototype and identify key features	Test group and research group: Community nurses and certified nursing assistants, including one designated coach (*n* = 7). Representatives of the research group (*n* = 3)	Strengths and weaknesses of the first prototype Key features for effective reflection and the use of the reflection method in clinical practice
Design phase 2 (refine)	To produce an overview of key features of the test meetings To probe work formats for the design group	Research group: Representatives of the research group (*n* = 3) Research group (*n* = 6)	An overview of the key features Appropriate design methods and materials
	To create more links between practice and the guideline recommendations	Design group and research group: Community nurses (*n* = 4), certified nursing assistants (*n* = 4), patient representatives (*n* = 1). Research group representatives (*n* = 3)	Prototype 2 of the reflection method Manual for prototype 2
Test phase 2	To test and evaluate the second prototype and identify key features	Test group and research group: Community nurses and certified nursing assistants, including one designated coach (*n* = 7). Representatives of the research group (*n* = 3)	Strengths and weaknesses of the second prototype Key features for effective reflection and the use of the reflection method in clinical practice
Design phase 3 (modify)	To present the final design and to modify it where necessary	Design group and research group: Design group (*n* = 9) Representatives of the research group (*n* = 3)	Final reflection method Manual for the conduct of the final reflection method

### 3.5 Study procedures

#### 3.5.1 The cyclical process

**FIGURE 1:**
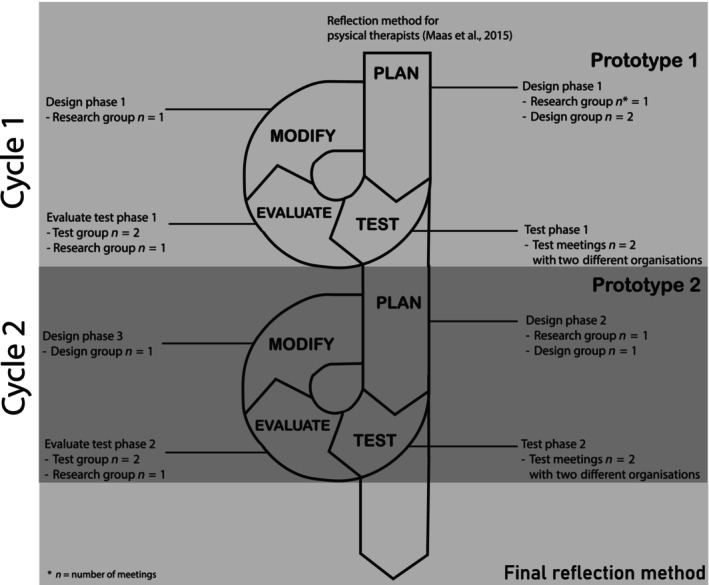
Schematic overview of the study: 15‐month process of two cycles.

This study involved a 15‐month cyclical process, between April 2022 and June 2023. This cyclical process aligns with the iterative model proposed by McKenney and Reeves (2012), which incorporates three core processes: (1) analysis and exploration, (2) design and construction and (3) evaluation and reflection (McKenney & Reeves, 2012). These processes were ensured in the cyclical process of this study through iteratively using design and test phases. In the design phases, the focus was on exploring, developing and reflecting in collaboration with the co‐participants, in the test phase it was on testing and evaluating the method. This 15‐month cyclical process comprised three design phases and two test phases (see Figure 1), and each phase lasted for 3 months.

Three groups worked closely together: a design group (*n* = 9) existing of community nurses and certified nursing assistants and patient representatives, four test groups including six participants each (community nurses and certified nursing assistants) and the research group (NV, MM, MH, MA, HK, PW). Four out of five design meetings took place online to save travelling time, in line with the preferences of the participants. All eight test meetings took place at the location of the participating organization and were supported by a coach to facilitate the reflection process.

The research group consisted of experts in the field of guideline development, community care, nursing research, educational research and reflective practice. The research group monitored the development process. Members of the research group (MH, MM and NV) guided the design and test groups closely.

#### 3.5.2 Design phase 1 (prototype 1)

The previously developed reflection method for implementing the reporting guideline for community nurses and certified nursing assistants served as the basis for designing the current method (Vullings et al., Submitted).

The first design phase consisted of a 2‐h meeting in which the design group with the researchers (NV, MH and MM) developed prototype‐1 of the reflection method for implementing the Informal Care guideline. The cultural probe method and the method of context mapping were used to generate ideas. The cultural probe method was used to stimulate creativity and to elicit the preferences and desires of professionals regarding the adequate provision of care by informal caregivers as well as good reflection practice (Burrows et al., 2015; van't Veer et al., 2020; Wherton et al., 2015). Context mapping was used to identify what the reflection method should yield for the community nurses, certified nursing assistants and informal caregivers (Sanders & Stappers, 2012; Visser et al., 2005). During the second design meeting, the empathy mapping tool was used. An empathy map is a tool for empathizing with and explaining what the user feels, hears and does (Woods et al., 2019). It seemed like an effective method to introduce the participants' client cases. This tool was adapted by three researchers (MH, MM and NV) to clarify what the informal caregivers feel, hear and do, according to the participating community nurses and certified nursing assistants. The first design phase resulted in prototype 1 of the reflection method.

At the end of this first design phase, the researchers (MH, MM and NV) prepared a program manual for the participants and the coaches, describing prototype 1 of the reflection method and the steps to be taken before, during and after the test meeting.

#### 3.5.3 Test phase 1

The coaches were trained at the start of the test phase by the research team (MM and NV), who are professional coaches. The initial training lasted 1.5 h.

The first part of the training focused on the sequential stages of the developed reflection method. The remaining time was used to practice these steps in a simulated setting.

Two test groups from different home care organizations tested prototype 1. Two hours were scheduled for each test meeting with the six participants. Meetings were facilitated by a coach. After the test meeting was finished, the experiences of participants were evaluated to identify the strengths and weaknesses of the first prototype.

#### 3.5.4 Design phase 2 (prototype 2)

The second design phase consisted of a 2‐h meeting in which the design group (*n* = 9) with the researchers (NV, MH and MM) identified how the prototype could be improved based on the evaluation results of the first test phase. An important outcome central to design phase 2 was the connection between the reflection method and the actual guideline. In test phase 1, participants did not make the link between their practical experiences with caregivers and the guideline recommendations. They needed more guidance to make this transfer. During design phase 2, therefore, a method based on the principles of brainwriting (van't Veer et al., 2020) was used to explore ideas to improve the connection of the method with the actual guideline. Brainwriting is an idea‐generation technique in which participants write down their ideas about a particular question in a few minutes (van't Veer et al., 2020). This technique initially involves no talking, which ensures that participants write down their own ideas without being influenced by other participants. The written text from each participant is forwarded to the next participant, who is asked to respond or elaborate on the idea (Steenbruggen et al., 2023; van't Veer et al., 2020). At the end of the brainwriting session, the group discussed which ideas were most appropriate and which ideas they were most energized by. It appeared that a practical tool with pictograms and the use of reflective questions could be useful in challenging participants to establish a stronger connection with the guideline. With the knowledge gained, the research team (NV, MH and MM) improved prototype 1, leading to prototype 2.

#### 3.5.5 Test phase 2

In the second test phase, the other two (of four) participating organizations tested prototype 2. In prototype 2, video feedback and a designed reflection game board with icon cards were tested. Again, 2 h were scheduled for both test meetings with six participants and a coach, and the strengths and weaknesses of prototype 2 were evaluated afterwards.

#### 3.5.6 Design phase 3 (final reflection method)

Three researchers (NV, MH and MM) from the research group discussed the data gained from the design and test meetings and suggested modifications to prototype 2. During the third meeting with the design and research group, these modifications were presented and discussed with all participants to reach consensus on the final reflection method.

### 3.6 Data collection

All meetings of the design group and test groups were video recorded. Informed consent was obtained from each participant. Following the meetings, the recordings were stored in a secured locked data cloud at the HAN University of Applied Sciences (Nijmegen) that only the researchers (NV, MH and MM) had access to. The video recordings of the design meetings lasted between 100 and 130 min and test meetings 100 and 120 min.

At least two researchers (MH, MM and NV) were present at each design and test meeting and afterward made notes of their individual observations of participant behaviours during the conduct of the reflection method and the evaluation.

### 3.7 Data analysis

All data from the design and test meetings were iteratively collected and analysed so that relevant information for the improvement of the design could be identified and used to modify the reflection method for the next design phase (Armstrong et al., 2022; Zidoun et al., 2022).

#### 3.7.1 Analysis of the notes

The first author (NV) compared the notes of MH and MM, and differences relevant to the design of the reflection method were discussed. Consensus between the three researchers was reached for the notes for which there seemed to be a difference in interpretation. When the notes were relevant to the development of the reflection method, these parts of the video recordings were studied again and discussed to better understand the behaviour of the participants and the group dynamics (Rania et al., 2021). After conducting observations during the test meetings, the three researchers (NV, MH and MM) engaged in discussions to determine what worked and what did not work within the reflection method. These aspects were used as predefined codes in the thematic analysis of the video recordings to identify the key features of the reflection method.

#### 3.7.2 Analysis of the video recordings

An external company, a representative of which signed a non‐disclosure agreement, transcribed the video recordings non‐verbatim. To identify the key features and themes of the reflection method, the transcripts were analysed using thematic analysis, as suggested by Braun and Clarke (2006).

The first transcript was independently studied and coded by the first author (NV) and two other researchers (MH and MM) in Atlas TI 22. They applied initial codes to the data relevant to the reflection method design and its key features. These codes were compared by the researchers with the predefined codes derived from their observations. They critically reflected on their perspectives and biases as researchers, using reflexivity and independent coding to enhance the trustworthiness of the study findings (Belotto, 2018; Humphreys et al., 2021; Malterud, 2001). By iteratively comparing codes and merging codes into higher code categories, they finally identified themes. These themes were related to a set of preliminary identified key features of the reflection method.

After the final test meetings, all video and observation data were used for triangulation. Preliminary key features were compared, conceptualized, and reframed, resulting in a final set of key features and themes.

### 3.8 Ethical considerations

This study adhered to the ethical principles of the Declaration of Helsinki. Ethical approval for this study was granted by the local ethics committee (Ethical Research Committee, ref.no REDACTED). Participation in this research was voluntary and the researchers maintained the participants' right to withdraw from the research.

### 3.9 Rigor and reflexivity

The study was reported using the Consolidated Criteria for Reporting Qualitative Research (COREQ). The research group consisted of experts in the field of guideline development, community care, nursing research, educational research and reflective practice.

## 4 RESULTS

### 4.1 Socio‐demographic characteristics

Twenty‐seven community nurses and certified nursing assistants participated in the study. Table 2 presents the background characteristics of the community nurses and certified nursing assistants.

**TABLE 2:** Characteristics of the study population.

Characteristics	*n* (%)
Total no. of participants	27
Gender	
Male	1 (3.7%)
Female	26 (96.3%)
Working hours	
<16 h	0 (0%)
16–24 h	22 (81.5%)
25–32 h	3 (11.1%)
>32 h	(7.4%)
Working experience	
0–3 years	5 (18.5%)
4–6 years	5 (18.5%)
7–10 years	3 (11.1%)
11–20 years	6 (22.2%)
>20 years	8 (29.6%)
Educational level	
Registered community nurse	10 (37.0%)
Registered nurse	7 (25.9%)
Certified nursing assistant	10 (37.0%)

### 4.2 Themes and key features

Five main themes were identified from the data. From these themes, nine key features for the reflection method emerged from the data. Table 3 presents all themes and key features. The themes and key features are elaborated upon below.

**TABLE 3:** Main themes and key features of the data.

Theme (*n* = 5)	Key features within the theme (*n* = 9)
The group	Limit groups to a maximum of six participants to allow varied input. Include participants from different teams, varying in working experience and educational levels. Form pairs of participants with different educational levels to support and assist each other during the preparation of the meetings.
Reflective triggers	Stimulate participants to express their thoughts about the informal caregivers' situation (awareness). Use video feedback for participants to reflect on their communication skills. Encourage enthusiasm by employing a reflection game that is simple and concrete, thus making it appealing to all participants.
Knowledge about clinical guidelines	Continuously make the connection with the actual guideline in discussing client cases.
The coach	Train the coach to be a process facilitator.
Preconditions	Meet organizational and contextual conditions for successful implementations.

#### 4.2.1 The group

During the test phases, three key features related to the group composition were identified: group size, participants with different backgrounds and pairs of participants. The video recordings and observations of the researchers showed that six participants were a desirable number to achieve sufficient depth, have fruitful group interaction and have enough time for discussion. Observations and video recordings showed that a combination of participants from different teams and with different levels of education was inspiring. Mixed groups allowed for awareness of daily practice and new perspectives on clinical routines. The collaboration with participants from different educational levels inspired them to work together as a team and share responsibilities.

She [a community nurse from a different team] asked questions and came up with suggestions that I would not have thought of myself at all. Within your own team, you often create your same way of working. (R6)

When something is being developed or a project is started, usually only community nurses are involved, which often excludes the entire team. Besides, it is beneficial that we do this together, as we are collectively responsible for the care of our clients, across all work experiences and educational levels. (R17)

The pairs of community nurses and certified nursing assistants indicated that they regarded preparing the meetings together as pleasant and useful. It encouraged them to prepare for the meetings earlier and ensured that they discussed which steps could be taken to achieve predetermined goals with the informal caregiver. In addition, certified nursing assistants also expressed the need to prepare for the meetings together. They felt empowered by their colleague and entered the conversation with the informal caregiver more prepared. Furthermore, the participants indicated that they appreciated everyone's involvement and participation in discussing the presented case.

We all work with the same target group and recognize the stress and sadness that can sometimes accompany informal caregiving. Therefore, everyone is willing to discuss and take actions to prevent overload or provide support for the informal caregiver wherever possible. (R18)

Both community nurses and certified nursing assistants indicated that they appreciated the team effort even more because of the solo nature of their work in community nursing.

#### 4.2.2 Reflective triggers

Participants needed help to reflect on their behaviour regarding informal caregivers. Reflective tools such as an empathy maps, reflection games and video feedback were helpful to increase awareness of clinical performance and bridge the gap between guideline recommendations and professional practice.

Observations and video recordings showed that the participants perceived the empathy map as helpful in expressing their implicit thoughts about the informal caregivers' situation. The use of the empathy map stimulated reflection on the needs of informal caregivers and gave participants input for the conversation with the informal caregiver. The participants found video recording their conversations with informal caregivers intense yet beneficial. It enabled them to provide detailed feedback and deeply reflect on their interactions. This applied to both their own recordings and those of their colleagues. In addition, the researchers' observations and video recordings of conversations with informal caregivers highlighted how reviewing the footage increased participants' awareness of the importance of supporting the informal caregivers. It emphasized the importance of aligning discussions with the informal care guideline and assessing their communication skills in these interactions.

Participants initially had doubts and felt tense about recording themselves and involving informal caregivers. Yet they found it highly beneficial to review both their own and others' recordings. Analysing these videos helped them observe and reflect on their interactions with informal caregivers, fostering critical self‐reflection and collaborative assessment. This, in turn, enabled them to plan more concrete actions in their approach to caregiving.

I learned a lot from looking back at the video recording. It is also much more appealing when you see a recording of someone else—much more than when someone tells you how the conversation went. (R8)

During the testing phases, several benefits related to improving caring for the caregiver were identified. The researchers' observations and video recordings showed that participants gained an understanding of meaningful actions in caring for the informal caregivers by reflecting on their own behaviours. In addition, talking to the informal caregiver appeared to change their view of the informal caregiver's burden. In some cases, the overload was overestimated, in others underestimated. Participants realized the added value of a structured conversation with informal caregivers.

I had never been in contact with this informal caregiver before, but at the initial conversation, she appeared to have been extremely overburdened for a long time. (R13)

Some community nurses and certified nursing assistants had difficulty understanding the terminology and steps taken in the reflection method. Some indicated that terms such as ‘reflect’, ‘recommendations’ and ‘learning questions’ were unfamiliar to them. In addition, they felt that the reflection method consisted of many steps, making it challenging to translate it to their own practice. This was addressed in the final reflection method by using simpler language and reducing the reflection method to fewer but more concrete steps.

The certified nursing assistants are not often involved in projects like this. For me, this is completely new. I enjoy being able to participate and learn something about the informal caregivers … But I notice that many words are completely unfamiliar to me. Additionally, I also observe that community nurses think much faster, and everything is happening very quickly. I need more time to think about things. (R24)

The researchers' observations and video recordings revealed that the use of the developed reflection game in the first meeting had a positive effect on the group dynamics and the learning process. The game board, which was in the middle of the table, ensured that everyone was involved in discussing each case and was invited to participate.

It is fun to do it through a game. A game is practical, which also encourages people to actively participate. It immediately appeals because it looks simple. And that is something very important, nice and easy, not too complicated [participant laughs]. (R25)

The reflection game format generated enthusiasm among participants to explore the case of the informal caregiver. The reflective questions on the icon cards prompted deeper consideration of the caregiver's situation and provided exposure to measurement tools and recommended interventions from the guideline. This facilitated discussions about applying these tools and interventions to the chosen case. Some participants suggested aligning the game content more closely with the guideline by incorporating additional guideline‐based cards.

#### 4.2.3 Knowledge about clinical guidelines

The evaluation of the test groups showed that almost none of the participating community nurses and certified nursing assistants was familiar with the term ‘guideline’. They knew the content of the Informal Care guideline through this project, but they were not familiar with the concept of a guideline and how this could support their daily practice. They therefore did not make the connection between the reflection method, the guideline, and daily practice.

First of all, I am curious if someone can tell what a guideline generally is. (R22)

No idea. (R28)

Coming close to. (R25)

To follow something, to try to create something. (R26)

The empathy map, video recordings, and the reflection game serve as triggers to work more in line with the guidelines. Additionally, participants indicated that the coaches should pay more attention to the connection with the guidelines and discussing practice. Participants indicated that the coach could do this by more clearly stating and repeating the purpose of the meeting at the beginning and posing more reflective questions about the client's case in relation to the guideline. The coaches indicated that additional training is necessary to teach the coach these skills.

The coach should play a role to make the connection between the guideline and practice. This can be done by asking critical questions and introducing and using the guideline more during the meeting. (R9)

#### 4.2.4 The coach

During the study, one key feature related to the role of the coach was identified: training the coach to be a process facilitator. During the meetings, the coaches experienced the role of facilitator as pleasant and challenging. Coaches indicated being focused on following the steps in time, although they regarded the steps of the reflection method as being clearly outlined and they felt that they had sufficient time. They found it challenging to stick to their role of coach, however. One coach had some experience with coaching, while the rest of the coaches had no knowledge of coaching before receiving the training.

The final design meeting and the critical reflection of the researchers on the analysis of the test meetings indicated that basic knowledge of the concepts of guidelines and evidence‐based working is an important prerequisite for an effective reflection method. Thus, an important feature of the reflection method is that the coach should be trained to gauge and respond to prior knowledge of guidelines to adequately link to the actual guideline.

#### 4.2.5 Preconditions

##### Face‐to‐face meetings

The first part of the study took place during the COVID‐19 pandemic when the meetings were conducted digitally. Video recordings and the researchers' observations showed that participants in digital meetings were less engaged with each other than they were in face‐to‐face interactions. Face‐to‐face meetings showed greater engagement, as participants made eye contact and responded actively to each other. Most participants preferred face‐to‐face meetings because of the ability to better interpret non‐verbal cues and because they experienced a greater sense of togetherness. A few participants acknowledged the practical benefits of digital meetings.

I prefer face‐to‐face meetings because feelings and emotions can be expressed better. It's more natural and really educational. You interpret things differently when you see them in real life. (R2)

##### Digital support

A final need identified in the study is digital support during reflection method meetings. The video recordings of the test meetings revealed that an effective sound system and projector are a requirement to play the videos of the conversation with the informal caregivers intelligibly. In addition, the evaluations of the test meetings revealed that the electronic health record needs to be made more conducive to the implementation of the Informal Care guideline by integrating the various tools and measurements of the guideline.

It would be nice if the measuring instruments from the guideline could also be found in a client's digital file so that I could access them immediately if necessary. (R9)

##### Time

An important component that emerged from both the video recordings and the observations was the time between the two meetings. Participants indicated that there should be sufficient time between the two meetings to prepare themselves properly. A period of 6 weeks was regarded as most desirable because of workload and scheduling. A period of 6 weeks should make it possible to meet and schedule a meeting with the informal caregiver.

#### 4.2.6 Final design

**FIGURE 2:**
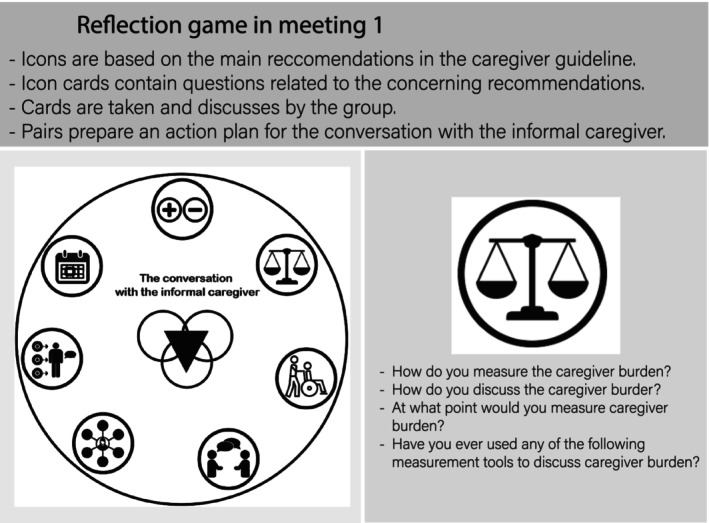
The final reflection game with one of the icon cards in the first meeting.

The final design of the reflection method is shown in Table 4. For the participants, the reflection method consists of a preparatory task, two meetings, in which they formulate personal goals and actions to be undertaken after each test meeting. During the first meeting, the main focus is the reflection on informal caregivers' cases using the game element, in which they reflect on the main topics (icon cards) of the informal care guideline. The reflection game board with the icon cards used in this meeting is presented in Figure 2. During the second meeting, participants reflect on short video recordings of their conversations with the informal caregiver.

**TABLE 4:** Final reflection method to support the implementation of the informal care guideline for community nurses.

Reflection method to support implementation of the informal caregiver guideline in community nursing	
*Actions of pairs before meeting 1*	*Actions of pairs before meeting 2*
Choose an informal caregiver. Gain permission. Describe the core of the case. Complete the empathy map.	Have a conversation with the informal caregiver and video record it. Watch the video recording and pick a 3–5‐min clip to show in the meeting. Bring a question for the group about the recording.
*Steps in meeting 1*	*Steps in meeting 2*
The coach opens the meeting and reviews prior knowledge of guidelines. Pair 1 briefly explains their case and shows the empathy map. The group asks questions to clarify the case. Repeat steps 2 and 3 for pairs 2 and 3. The coach explains the next step with the reflection game. Each pair, one by one, takes an icon card and reads the questions. The group discusses the questions. The pairs think about the next steps in the conversation with the informal caregiver and tell the group what they are. The coach evaluates the meeting with the group (process and yields/gains) and summarizes the reflection method steps to be taken after the meeting.	Pair 1 says what actions were undertaken over the past weeks and the results. Pair 1 explains why this video clip was chosen. The video clip is shown. The group gives feedback on the video clip. The pair responds to the feedback. Pair 1 introduces their question related to the video clip. Each participant gives advice. The coach asks questions to help the pair formulate a new goal and the next steps to be undertaken. Repeat steps 1–7 for pairs 2 and 3. The coach evaluates the meeting with the group (process and gains) and summarizes the steps to be undertaken after the meeting.
*Actions of pairs after meeting 1*	*Actions of pairs after meeting 2*
Each pair formulates 1–3 learning goals and an action plan and acts accordingly.	Each pair evaluates the progress on the previously formulated learning goals and actions.

## 5 DISCUSSION

This study developed, tested and evaluated a reflection method to support the implementation of the national Informal Care guideline for community nurses and certified nursing assistants. The close collaboration of end‐users with the research team resulted in a reflection method with two 2‐h meetings that matches the learning needs, preferences and specific context of this heterogeneous group. Five main themes were identified from the analysis: the group, reflective triggers, knowledge about the guidelines, the coach and preconditions. These themes were linked to nine key features representing the building blocks of the reflection method. The key features were group size, participants with different (educational) backgrounds, pairs of participants, expressing thoughts, video feedback, reflection game, making the connection with the guideline, the coach as a process facilitator and meeting organizational and contextual conditions for implementation.

Participants regarded it as valuable to review both their own and others' video recordings, focusing on visual aspects and the content of what was said. Sellitto et al. (2016) confirm this finding and show that watching their own video recordings is helpful for students in their self‐reflection and helpful in discussing actions or communication skills with other students (Sellitto et al., 2016). In healthcare, research shows that video feedback facilitates reflection and self‐directed learning and thus improves the ability to develop new skills (Halim et al., 2021). In addition, it allows people to visualize their strengths and weaknesses and thus gain insights (Akcan, 2010; Kam et al., 2019). In contrast, the research of Yoong et al. (2023) indicates that peer feedback and video feedback are equally effective in enhancing clinical competencies (Yoong et al., 2023). In their study, peer video feedback was experienced as considerably more stressful for nurse students. This aligns with our study in which it was observed that initially creating the video recording was perceived as stressful. Nonetheless, the participants expressed afterward that reviewing the recording contributed to their learning.

An unexpected outcome during the study was that participants' familiarity with guidelines in general was limited. There was a noticeable lack of knowledge and intrinsic interest in both guidelines in general and the specific Informal Care guideline under consideration. Ample research has investigated the knowledge of physicians and nurses regarding the specific guidelines that they should work with. Many of these studies show that care professionals have only limited knowledge about these guidelines (Jun et al., 2016; Patell et al., 2019; Wang et al., 2020). The Consolidated Framework for Implementation Research (CFIR) indicates that knowledge about the intervention or guideline is an important determinant of successful implementation (Damschroder et al., 2022). Previous research has shown that guidelines are often difficult to implement in nursing practice, leading to a lack of adherence (Arts et al., 2016; Lugtenberg et al., 2009; Rycroft‐Malone et al., 2016). The development of clinical guidelines does not guarantee acceptance and adherence in practice. A previous review examined the barriers to guideline implementation in healthcare (Fischer et al., 2016). These barriers were related to various factors such as personal factors (knowledge and attitude), guideline‐related factors (evidence, applicability in the specific context and complexity) and external factors (resources in the organization, social norms and values) (Fischer et al., 2016). Important factors such as knowledge, attitude and organization conditions are consistent with this study. Another important facilitator for implementation consistent with our study is ownership among nurses (Candas et al., 2016; Weis et al., 2014). Our study indicated that both community nurses and certified nursing assistants experienced it as very pleasant and motivating to be involved in the development of the method. This made them feel acknowledged and recognized for the value they added in providing care. Not involving community nurses and certified nursing assistants appears to be a barrier to the implementation of an innovation.

This study shows that both community nurses and certified nursing assistants lack knowledge and use the guidelines infrequently. Community nurses and certified nursing assistants rarely consult guidelines because of the size of these documents and the language used (Arts et al., 2016; Fischer et al., 2016; Flottorp et al., 2013). A possible solution to this would be for the information from the guideline to be presented in a more concrete and practical manner. Discussing guideline recommendations by using a game that includes questions based on these recommendations seems to encourage community nurses and certified nursing assistants to reflect on the care given. Participants were enthusiastic about the game element, and it challenged them to discuss the questions in the game. This is consistent with research by Muijen (2001) who found that the game element has the ability to engage the participants.

Learning through fun, a principle of play‐based learning, was also exemplified in this study, as participants learned how to apply a guideline using a concrete case. Perrotta et al. (2013) describe the principles of game‐based learning: intrinsic motivation, learning through fun, autonomy and experiential learning (Perrotta et al., 2013). Finally, this study highlights that certified nursing assistants in particular appreciate being part of quality‐improvement initiatives mainly because they do not usually participate. Having their own role in this game, through which the entire group learns, reinforces their autonomy (Jacobs, 2000).

The reflection method aims to create awareness and change behaviour regarding acting with guidelines in community nursing. However, without the explicit guidance of a trained coach the connection between the actual guideline and practice is missed, and trying out recommendations and tools recommended in the guideline remains limited. The role of the coach is therefore important. Leigh et al. (2019) showed that training for the role of coach is crucial. The coach assists participants in learning by asking reflective questions and assuming an independent role (Leigh et al., 2019). This finding is consistent with the present study, where nurses were helped to learn and attention was paid to their own development. Because nurses see tangible results in terms of personal goals and see the contribution they make to care within an organization, they find their work more meaningful (Downey, 2003). Several factors are important for the coach to be effective: creating safety and openness within the group and demonstrating leadership qualities and intervening when necessary in addressing issues and problems to get to the core of the problem (Narayanasamy & Penny, 2014). This finding is in line with the results of this study. Additionally, this study showed that it was crucial for the coach to have basic knowledge of guidelines. This finding is in line with what was experienced by the coaches in this study and observed by the researchers.

### 5.1 Strengths and limitations

A major strength of this study is the research design. The design‐based approach allowed the reflection method to be developed with the end‐users, ensuring a match with their nursing practice (Ackerman et al., 2008; Wang & Hannafin, 2005). The reflection method is a result of a co‐creation process between researchers and end‐users (Armstrong et al., 2022). Obtaining participants' input and feedback on the reflection method adds validity to the results by ensuring that the participants' own beliefs and perspectives are represented and not constrained by the researchers' own agenda or knowledge (Popay & Williams, 1998). The end‐users had different demographic characteristics, such as age, education level and years of work experience. By comparing this group's evaluation to that of the national nursing community (Francke et al., 2017), one can conclude that the participants in this study were representative of the group of community nurses and certified nursing assistants, ensuring the link to their daily practice.

The second strength of this study is that all the data from the design and test meetings were iteratively collected and analysed, resulting in relevant information and directions for improvement of the prototype, which was used in the next design phase (Ackerman et al., 2008; Wang & Hannafin, 2005). Moreover, triangulation of data resources strengthened the credibility of the results (Carter et al., 2014; Noble & Heale, 2019).

A design‐based approach might also be regarded as a limitation because it primarily focuses on designing a method that is valid in a specific context, making it challenging to generalize to another context (van't Veer et al., 2020). Therefore, we established key features that are expected to be transferable to other contexts and other guidelines. Another limitation of the study is that only one patient representative participated. This study concerned a reflection method for care professionals and how they can be supported to work according to the informal care guidelines in daily practice. We could have involved more patients and informal caregivers in the development of the reflection method by, for example, asking for feedback on the perceived benefits of the healthcare professionals' actions, especially because understanding the effective elements in the relationship between the nurses and the client is crucial for nurses' motivation (Guay et al., 2000; Ryan & Deci, 2000).

### 5.2 Implication for practice and further research

Reflection through a method containing a game element and video feedback provides important benefits for learning and better applying guidelines in clinical practice (Asselin & Fain, 2013; Contreras et al., 2020; Halim et al., 2021; Paget, 2008). It is therefore recommended that a reflection method is used for the implementation of guidelines to enhance care according to the guidelines that are to be implemented within community care organizations. To properly implement the reflection method within these organizations, it is important to further examine the barriers and facilitators and develop adequate implementation strategies.

This study indicates that community care professionals have little explicit knowledge about guidelines in general. Knowledge about guidelines needs to be incorporated into a reflection method to support guideline implementation. In the future, a design‐based approach could be employed to discover what community nurses and certified nursing assistants need to acquire more basic knowledge about guidelines and evidence‐based practices.

As well as using a reflection method, it is important to make the guidelines more accessible for community nurses and certified nursing assistants. This can be achieved by creating summaries, videos and mobile phone apps. Finally, in further research, it is crucial to examine on a larger scale both the effect on the community nurses and certified nursing assistants and the impact of this reflection method on supporting informal caregivers from the perspective of the informal caregivers.

## 6 CONCLUSION

We developed an evidence‐ and practice‐based reflection method for community nurses and certified nursing assistants to support the implementation. By involving community nurses and certified nursing assistants, we developed the method to closely match their needs and preferences.

The reflection method consists of two 2‐h meetings with up to six participants and a coach. The participants need guidance to make the transition from guideline recommendations to practice. The establishment of this transition and the reflection on care is facilitated by reflective triggers, namely expressing thoughts about the informal caregivers' situation, using video feedback, and a simple and concrete reflective game.

## AUTHOR CONTRIBUTIONS

All authors have agreed on the final version and meet at least one of the following criteria: substantial contributions to conception and design, acquisition of data or analysis and interpretation of data; drafting the article or revising it critically for important intellectual content.

## FUNDING INFORMATION

This work was supported by ZonMw, the Netherlands, 80‐87100‐98‐003.

## CONFLICT OF INTEREST STATEMENT

No conflict of interest has been declared by the authors.

## PEER REVIEW

The peer review history for this article is available at https://www.webofscience.com/api/gateway/wos/peer‐review/10.1111/jan.16156.

## Data Availability

Anomised data will be available on request.
